# Cumulative incidence of chronic health conditions recorded in hospital admissions from birth to age 16 in England: a cohort study using linked hospital and education administrative data

**DOI:** 10.23889/ijpds.v9i5.2485

**Published:** 2024-09-18

**Authors:** Matthew Jay, Lauren Herlitz, Jessica Deighton, Ruth Gilbert, Ruth Blackburn

**Affiliations:** 1 UCL GOS Institute of Child Health; 2 UCL and Anna Freud Centre for Children and Families

## Objectives

Monitoring the incidence of chronic health conditions (CHCs) in childhood in England using administrative data to derive numerators and denominators is challenged by unmeasured migration. We used a birth cohort design and different criteria for contact with health or education services to estimate the cumulative incidence of CHCs to age 16 years.

## Approach

We identified all births in Hospital Episode Statistics (HES) from 2002/3 to 2011/12, with follow-up to 2018/19 (maximum age 8 to 16 years), censoring on death, first non-England residence record or 16th birthday. Children must have linked to later HES records and/or the National Pupil Database (NPD), which provides information on all state-school enrolments. The cumulative incidence of CHCs was estimated to age 16 using diagnostic codes in hospital admission records. We also explored temporal variation. Sensitivity analyses varied denominator criteria.

## Results

Between 423,456 children (79.4%, of all HES-recorded births in 2002/3) and 618,054 (97.5% in 2011/12) linked to later HES or NPD records. The cumulative incidence of ever having a recorded CHC before age 16 among children born in 2002/3 or 2003/4 was 24.6%, ranging from 21% to 32% in sensitivity analyses. There was little temporal variation. At least 28% of children with any CHC had more than one body system affected by age 16. Multimorbidity rates rose with later cohorts.

## Conclusions

Approximately one quarter of children are affected by CHCs, but varies depending on how the denominator is defined. More accurate estimation of the incidence of CHCs requires a dynamic population estimate.

